# Coordination Between Phloem Loading and Structure Maintains Carbon Transport Under Drought

**DOI:** 10.3389/fpls.2022.787837

**Published:** 2022-02-17

**Authors:** Ryan C. Stanfield, Megan K. Bartlett

**Affiliations:** Department of Viticulture and Enology, University of California, Davis, Davis, CA, United States

**Keywords:** carbon transport, drought, phloem resistance, phloem loading (apoplasmic, symplasmic), viscosity limit, phloem anatomy, molecular regulation

## Abstract

Maintaining phloem transport under water stress is expected to be crucial to whole-plant drought tolerance, but the traits that benefit phloem function under drought are poorly understood. Nearly half of surveyed angiosperm species, including important crops, use sucrose transporter proteins to actively load sugar into the phloem. Plants can alter transporter abundance in response to stress, providing a potential mechanism for active-loading species to closely regulate phloem loading rates to avoid drought-induced reductions or failures in phloem transport. We developed an integrated xylem-phloem-stomatal model to test this hypothesis by quantifying the joint impacts of transporter kinetics, phloem anatomy, and plant water status on sucrose export to sinks. We parameterized the model with phloem hydraulic resistances and sucrose transporter kinetic parameters compiled from the literature, and simulated loading regulation by allowing loading rates to decline exponentially with phloem pressure to prevent excessive sucrose concentrations from inducing viscosity limitations. In the absence of loading regulation, where loading rates were independent of phloem pressure, most resistance values produced unrealistic phloem pressures owing to viscosity effects, even under well-watered conditions. Conversely, pressure-regulated loading helped to control viscosity buildup and improved export to sinks for both lower and higher resistant phloem pathways, while maintaining realistic phloem pressures. Regulation also allowed for rapid loading and export in wet conditions while maintaining export and viable phloem pressures during drought. Therefore, we expect feedbacks between phloem pressure and loading to be critical to carbon transport in active-loading species, especially under drought, and for transporter kinetics to be strongly coordinated with phloem architecture and plant water status. This work provides an important and underexplored physiological framework to understand the ecophysiology of phloem transport under drought and to enhance the genetic engineering of crop plants.

## Introduction

The phloem is the “enigmatic central banker” that appropriates and transports carbon from the photosynthesizing sources to the carbon-consuming sinks (Ryan and Asao, 2014). For the plant to “cash-out” the carbon-rich phloem sap for growth, respiration, or storage, the phloem must maintain a sufficient pressure gradient from source to sink to drive bulk flow (Münch, 1930; van Bel, 2003). The phloem builds pressure at the source by drawing in water from the xylem, making plant water status important to carbon transport. A wide range of xylem and stomatal traits have been linked to maintaining hydraulic function under water stress and adapting plants to dry environments (Meinzer et al., 2009; Bartlett et al., 2016). However, the traits that confer phloem drought tolerance by maintaining pressure gradients for carbon transport under water stress are not well understood, due to the technical difficulty of measuring phloem transport *in vivo* (Jensen et al., 2016; Savage et al., 2016).

Experimental constraints have made modeling approaches crucial to assess the impacts of phloem traits and plant hydraulics on phloem transport (e.g., Thompson and Holbrook, 2003a,b; Hölttä et al., 2006, 2009; Jensen et al., 2012). Many models have demonstrated an important role for the coupling of phloem sucrose loading rate with photosynthesis to maintain phloem transport in response to drought stress (Daudet et al., 2002; Hölttä et al., 2009; Nikinmaa et al., 2013; Huang et al., 2018). However, for active loading species, photosynthetic regulation of phloem transport may not be necessary, as phloem loading rates may be modulated in response to environmental conditions through molecular regulation of sucrose transporters (e.g., Xu et al., 2018, 2020; Bush, 2020). This added regulatory pathway is important to study, because up to 42% of species surveyed utilize an active sugar loading mechanism (Rennie and Turgeon, 2009), including economically important crops such as celery, tobacco, spinach, tomato, cotton, sunflower, wheat, and grapevine (Kuo et al., 1974; Davies et al., 1999; Rennie and Turgeon, 2009; Muller et al., 2014). Active-loading species use specialized transport proteins to load sugar into the phloem. Transgenic studies have tested upregulating these transporters as a strategy to improve crop performance, but with mixed success; these manipulations improved vegetative growth and grain yield in some species and not others (Leggewie et al., 2003; Dasgupta et al., 2014; Wang et al., 2015; Lu et al., 2020). A greater understanding of the impact of active transport mechanisms on phloem function is needed to inform crop improvement efforts (Lawlor, 2013; Braun et al., 2014) and to better understand the ecological and evolutionary significance of this loading strategy (Savage et al., 2016). Thus, we conducted the first study to our knowledge evaluating the impacts of sucrose transporter kinetics and loading regulatory mechanisms, and the interactive effects of phloem anatomy, on sugar translocation under drought.

Sucrose loading into phloem (Lemoine et al., 2013) as well as transport long distance from source to sink (Hölttä et al., 2006, 2009; Zhou et al., 2020) is closely linked to drought. Drought stress impacts phloem transport by making it more difficult for water to enter the phloem. As water potential becomes more negative (i.e., drier) in the leaf xylem, the phloem is hypothesized to compensate by increasing the sugar concentration in the loading zone (Turgeon, 2010). This mechanism reduces the osmotic potential and strengthens the water potential gradient drawing in water from the neighboring xylem. This influx of water generates the high turgor pressure in the loading zone that powers source-to-sink carbon transport. However, this presents a conundrum, since greater sucrose loading and the resulting higher concentrations increase the viscosity of the phloem sap and, thus, the hydraulic resistance of the phloem (Nikinmaa et al., 2013; Sevanto, 2014, 2018; Salmon et al., 2019). In well-watered conditions, the sugar concentrations measured in many species are consistent with those modeled to be optimal for efficient flow (Jensen et al., 2013). However, a higher viscosity pathway (i.e., higher phloem pathway resistance) means that even more loading is needed to generate the pressure differentials required for transport. This can create feedback between loading and resistance that becomes untenable, causing transport to slow or even stop as the sap becomes too viscous to push down to the sinks, (i.e., “viscosity limitation”) (Hölttä et al., 2009; Nikinmaa et al., 2013; Sevanto et al., 2014).

In active loaders, membrane transporter proteins lining the phloem conduit (sieve element/companion cell complexes) membranes load in sugars from the surrounding extracellular space (apoplasm) (Rennie and Turgeon, 2009). Unlike passive (symplasmic) loaders, this mechanism decouples loading rates from the concentration gradient between the mesophyll and phloem loading complex (Lalonde et al., 2004; De Schepper et al., 2013; Schulz, 2015; Milne et al., 2018; Rockwell et al., 2018). This mechanism could reduce constraints on phloem transport by allowing loading to be regulated independently from photosynthesis (Nikinmaa et al., 2013; Huang et al., 2018; Xu et al., 2018), and is hypothesized to produce the higher phloem concentrations and pressure potentials observed in active loaders (Turgeon, 2010). These higher pressures could be advantageous in discouraging phloem-feeding pests or supporting faster growth rates (Savage et al., 2016), but potentially increase the risk of viscosity limitation during drought. However, active loading could also prove beneficial to translocation under drought, by tightly regulating sucrose transporter activity to increase phloem osmotic strength, while preventing viscosity limitations.

Sucrose loading transporters (SUTs or SUCs) are under dynamic regulation, especially in response to environmental stress (Ainsworth and Bush, 2011; Bush, 2020). This is evidenced by their rapid degradation in the loading complex membrane, with a half-life as short as 4 h (Liesche et al., 2011a). Under water stress, SUT transcript abundance can be upregulated (Ibraheem et al., 2011; Xu et al., 2018) or the transporters can be stabilized to prevent their breakdown from the plasma membrane (Ma et al., 2019), which would increase loading and strengthen the gradient for water uptake from the xylem. Alternatively, other experiments have shown high leaf sucrose concentrations to downregulate phloem loading rates (Chiou and Bush, 1998), pointing to a potential mechanism for active loaders to avoid viscosity limitations. The signal driving this dynamic regulation is unknown, but could be phloem turgor pressure, as loading rate has been demonstrated to respond to sieve tube pressure (Smith and Milburn, 1980). Moreover, phloem turgor has been hypothesized to trigger a hormonal signaling cascade that alters transporter expression or post-translational modification of sucrose transporters (Patrick et al., 2001). Thus, it is plausible that a mechanistic relationship regulates sucrose loading in a turgor-dependent manner, but the consequences for phloem function have yet to be explored. V_*max*_ (the maximum transport rate in Michaelis-Menten formalism) has only been measured for phloem loading proteins in less than six species (Cataldo, 1974; Kuo et al., 1974; Sovonick et al., 1974; Fondy and Geiger, 1977; Wimmers and Turgeon, 1991; Weise et al., 2000; Borstlap and Schuurmans, 2004; Thompson and Wolniack, 2008), while the functional impacts of variation in V_*max*_ and its regulation in the context of viscosity limitation and drought are unknown. It is also unknown whether an active loading mechanism may aid in preventing detrimental viscosity build-up through the dynamic down-regulation of sucrose loading proteins.

**FIGURE 1:**
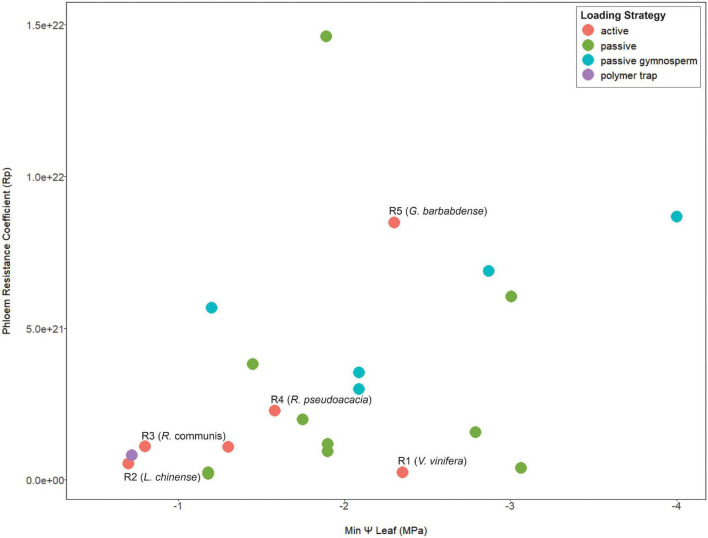
A meta-analysis scatterplot for phloem resistance coefficients and minimum mid-day leaf Ψ that show no association between these two parameters in terms of loading type. Points are color coded by species phloem loading strategy. The species used in the model simulations are indicated from R1–R5 in order of increasing pathway resistance. Anatomical data is from Liesche et al. (2017) and scaled equally to obtain a phloem pressure of >0.6 MPa for all resistance values. Minimum mid-day leaf Ψ was obtained from Choat et al. (2012) and Bartlett et al. (2016). See Supporting Information Table 1 for data and references used for loading types. Here we assume Gymnosperms are passive loaders, although a systematic study of this taxa for loading mechanism is still warranted (Liesche et al., 2011b; Liesche, 2017).

Since viscosity limitation is caused by excessive resistance along the transport pathway, the structural resistance of the sieve tube is expected to impact the risk of transport failure. Both the sap viscosity and the dimensions of the sieve tube contribute to the vulnerability to viscosity limitation (Sevanto, 2014). More specifically, phloem anatomical properties such sieve tube diameter and end wall (sieve plate) porosity strongly impact phloem resistance (Hölttä et al., 2009; Mullendore et al., 2010; Jensen et al., 2012; Stanfield et al., 2019). Further, pathway resistance can suddenly increase due to callose accumulation (Esau et al., 1962) from insect or mechanical damage (Pickard and Minchin, 1992; Hao et al., 2008). In other words, conduits with higher resistance due to structure are more prone to viscosity limitation, especially in the presence of callose. However, phloem structural resistance (estimated from sieve tube anatomy) was not correlated with habitat water availability (Liesche et al., 2017); while many of those species were likely passive loaders, data we compiled also shows no relationship between loading mechanism, pathway resistance, and maximum drought stress (Figure 1). Viscosity limitation could potentially limit the maximum loading rate in a species with high structural resistance, selecting for coordination between phloem anatomy and transporter kinetics. Further, it can be hypothesized that active-loading species from environments with frequent drought could critically depend on downregulation of loading to control viscosity limitation, especially species with high structural phloem resistance.

**FIGURE 2:**
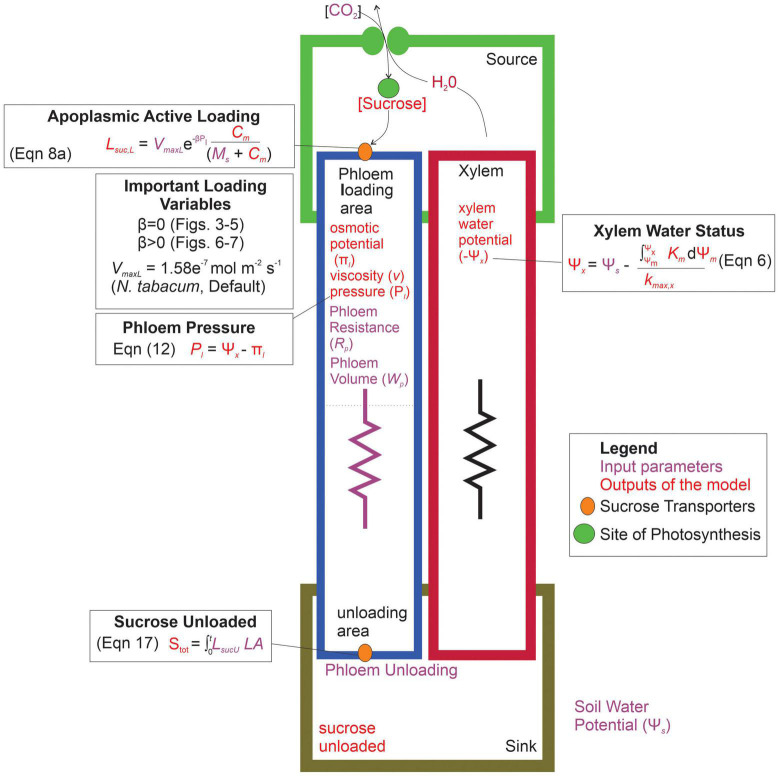
Parameterization and conceptual schematic of the water and sugar transport model. The model inputs are shown in purple, while the outputs of the model are in red. The model first produced a xylem water potential based on the input soil water potential. Sucrose concentrations are built in the leaf compartment (source), and actively loaded into the phloem through protein transporters. Loading variables that were altered in the different simulations are shown, which impact the resulting phloem specific outputs such as: phloem osmotic potential, viscosity, pressure, and the eventual sucrose exported to the sink area. Symbols: L_*sucL*_ = loading rate per unit leaf area, V_*maxL*_ = maximum rate of sucrose uptake, β = shape parameter for loading downregulation, *Pl* = phloem pressure in loading area, *C* = concentration of sucrose in source area, *M*_*S*_ = Michaelis-Menten constant for sucrose affinity, *S*_*tot*_ = total sucrose exported over the entire simulation, L_*sucU*_ = total unloading rate, *LA* = leaf area, Ψ_*m*_ = water potential of source area, k_*m*_ = hydraulic conductivity of mesophyll, k_*max,x*_ = maximum hydraulic conductivity of xylem.

Overall, our goal was to investigate the interactive effects of loading transporter kinetics and phloem anatomy on sugar transport across a range of water stresses. We predicted that the constraints of viscosity limitation to strongly coordinate phloem loading rates with phloem structural resistance and plant water stress. Specifically, this study addressed the questions: (1) what are the interactive effects of phloem anatomy and drought intensity (i.e., soil water potential) on sugar export by active-loading species? and (2) in scenarios where phloem resistance and drought induce viscosity limitation, how does changing loading transporter kinetics, including introducing pressure-coupled downregulation, impact sucrose export to sinks? We addressed these questions by integrating a stomatal-hydraulic model (modified from Bartlett et al., 2019) that calculated leaf water potentials and gas exchange rates from environmental and hydraulic trait inputs. We then combined this with a simple phloem transport model (De Schepper and Steppe, 2010) that calculated phloem pressure gradients and flow rates within a single sieve tube from leaf sucrose concentrations and water potentials, using the Michaelis-Menten enzyme formalism to represent active loading and unloading (Figure 2).

## Materials and Methods

### Model Overview

Conceptually, the model was divided into three components: plant water status, gas exchange and phloem transport (Figure 2). The plant water status component calculated the leaf mesophyll and xylem water potentials (Ψ_*m*_ and Ψ_*x*_) from an input soil water potential (Ψ_*s*_), gas exchange and hydraulics. The gas exchange component calculated photosynthesis from the stomatal aperture, which was determined from the mesophyll water status (Ψ_*m*_). The phloem transport component calculated a source – sink phloem sucrose concentration and pressure gradient generated from the Michaelis-Menten kinetics of loading/unloading sucrose and the water stress experienced by the phloem (Ψ_*x*_ or Ψ_*s*_).

### Model Assumptions

To simplify the model, we made the following assumptions:

(1) The simulated plant used active phloem loading and unloading, which captures the mechanisms driving crop production in many economically important species [e.g., grapevine (Zhang et al., 2006), tomato (Hackel et al., 2006), and kiwi (Gould et al., 2013)]. The unloading rates depended on the sucrose concentration in the phloem unloading zone, not the concentration gradient between the unloading zone and sink tissue. This also captures passive unloading dynamics for species that enzymatically convert unloaded sucrose to starch (Goeschl and Han, 2020), or use active uptake processes to remove sucrose immediately surrounding the phloem unloading zone into storage vacuoles (Saftner et al., 1983).
(2) Unloading did not constrain sucrose export, and all sucrose that reached the unloading zone was exported in the same timestep (i.e., the maximum unloading rate *V*_*maxU*_ >> *V*_*maxL*_). This assumption has some empirical support, as unloading fluxes from the roots of pea plants (*Pisum sativum*) are 95.8% higher than in active loading rates from this species (Wimmers and Turgeon, 1991; Liesche, 2017). Further, we expected unloading limitations to simply exacerbate viscosity limitations by pressurizing the unloading zone and reducing the turgor gradient for phloem transport. Thus, in this study, we prevented unloading from having a confounding effect on viscosity limitation by assuming V_*maxU*_ was 20% greater than V_*maxL*_ (see Supplementary Figure 1 on the consequence of loading limitation on phloem pressure).
(3) All sucrose produced by photosynthesis was available for loading into the transporting sieve elements (i.e., we do not model intermediary transport through the mesophyll or companion cells).
(4) Sucrose only entered/exited from the phloem loading/unloading zones, and the sucrose exported from the unloading zone arrives in sink tissue outside the sieve tube. Leakage of sucrose back out of the loading area by diffusion did not meaningfully impact sucrose export to sinks (Supplementary Figure 2).
(5) Phloem water potentials equilibrate with the source xylem, but we did not explicitly model water flow or volume.
(6) Total phloem resistance (R_*conduit*_) was calculated from sieve element structural resistance (*R*_*p*_) and phloem sap viscosity (*v*), which increased with sucrose concentration (R_*conduit*_ = Rp × v).
(7) Structural resistance was calculated from sieve element dimensions compiled from the literature for stem tissue in active loaders (Liesche et al., 2017; Jensen, 2019). These species were not measured for several factors that impact scaling from sieve element to whole-plant resistance, including total path length or anatomical variation along the path length (i.e., allometric scaling). Thus, these values should be considered plausible first-order estimates, rather than precise species-specific parameters.
  Instead, we scaled resistance from individual sieve elements (*Rse*; representing the lumen and sieve plates) to a whole-plant phloem pathway by multiplying with a scalar value (2.5e23) and normalizing by the length of each sieve element (*Rsel*):

(1) $$Rp=\frac{Rse\times 2.5e23}{Rsel}$$

Since phloem anatomical data to obtain a whole plant phloem resistance is only sparsely available, we selected a scalar value that produced reasonable phloem pressures for our baseline/default parameter values (Table 1) (i.e., a minimum phloem pressure in the loading zone (P_*l*_) ≥ 0.6 MPa) (see Supplementary Table 1 for *Rse* and *Rsel* values).
(8) Viable phloem pressures in the loading zone ranged from 0.6 to 2.4 MPa, which captures the range of pressures reported in the literature from empirical pressure probe measurements of source-adjacent phloem tissue (reviewed in Turgeon, 2010). Simulations which produced pressures outside this range were considered biologically unrealistic.
(9) Light was constant and non-limiting for photosynthesis.
(10) The water volume in the mesophyll changed over time, while the xylem was at steady state.
(11) Sucrose concentration in the mesophyll did not impact the water potential of the mesophyll.

**TABLE 1:** Description of mathematical symbols used in the model.

Symbol	Definition	Value (or units)	References
*af*	Apoplastic fraction inside the leaf	0.3	Bartlett et al., 2019
*ags*	Shape parameter relating gsΨ_50_ and Ψ_*m*_	2.0	Bartlett et al., 2019
*A*_*net*_	Carbon assimilation through photosynthesis	(μmol CO_2_ m^–2^ s^–1^)	
*c*_*a*_	Atmospheric CO_2_ concentration	400 Parts Per Million	Bartlett et al., 2019
*C*_*m*_	Concentration of sucrose in mesophyll	(M)	
*C*_*pL,U*_	Concentration of sucrose in phloem loading (L) or unloading (U) area	(mM)	
*E*_*s*_	Rate of sucrose leaving phloem loading area	(m s^–1^)	
*Fp*	Volumetric flow rate of phloem	(m^3^ s^–1^)	
*g*_*max*_	Maximum stomatal conductance	400 mmol m^–2^ s^–1^ (default)	
*g*_*s*_	Stomatal conductance	(mmol m^–2^ s^–1^)	
*g*_*s*_ Ψ_50_	Water potential of mesophyll at 50% stomatal closure	−1.5 MPa	Bartlett et al., 2016
*K*_*m,x*_	Hydraulic conductance of mesophyll (m) or xylem (x)	(mmol m^–2^ s^–1^)	
*K*_*max*_	Maximum hydraulic conductance of the leaf	20 mmol m^–2^ s^–1^ MPa^–1^	
*M*_*S*_	The affinity of the sucrose molecule to the SUT protein	3.3 mM	Borstlap and Schuurmans, 2004
*LA*	Leaf area	47.4 cm^2^	
*L*_*sucL,U*_	Loading rate of sucrose per unit tissue area for the loading (l) or unloading (u) area	(mol m^–2^s^–1^)	
*mm*	Molar mass of sucrose	342.3 g mol^–1^	
*P*_*l, u*_	Phloem pressure in loading (l) or unloading zone (u)	(MPa)	
*r*	Radius of phloem conduit	5.5e^–6^ m (tobacco)	Thompson and Wolniack, 2008
*Rc*	Gas constant	8.3 m^3^ pa K^–1^mol^–1^	
*R*_*conduit*_	Total resistance of the phloem conduit	(MPa s m^–3^)	
*R*_*L*_	Leaf respiration rate, assumes 12 C atoms in 1 sucrose	0.25 μmol C m^–2^ s^–1^	Bartlett et al., 2019
*RF*	Dimensionless value which determines if phloem is in water potential equilibrium with xylem	Dimensionless	Thompson and Holbrook, 2003b
*Rp*	Structural resistance coefficient of the phloem	See Table 2	Liesche et al., 2017
*RWC*_*m*_	Relative water content of the mesophyll	(%)	
*Sl*	Leak rate of sucrose in the phloem conduit	7.3e^–16^ m s^–1^	Edelman et al., 1971
*S*_*m*_	Moles of sucrose in apoplasmic space	(mols)	
*S*_*p,b*_	Mass of sucrose inside loading phloem (p) or unloading phloem (b)	(g)	
t	Run time of model	12 h	
*T*	Temperature	293 K	
*v*	Viscosity of phloem conduit sap	(MPa s)	
*V*_*maxL*_	Maximum rate of sucrose uptake (phloem loading)	1.58e^–7^ mol m^–2^ s^–1^ (default)	Borstlap and Schuurmans (2004) (tobacco)
*V*_*maxU*_	Maximum rate of sucrose export (phloem unloading)	*V*_*maxL*_ × 1.2 mol m^–2^ s^–1^ (default)	
*VPD*	Vapor pressure deficit of leaf	9.9e^–3^ (dimensionless)	
*W*_*p*_	Water volume inside phloem	3.57e^–11^ m^3^ (R1 and R2; default) 1.04e^–8^ m^3^ (R3) 1.37e^–8^ m^3^ (R4 and R5)	
α	Shape parameter for determining mesophyll conductance	2	Bartlett et al., 2019
ρ	Density of phloem sap	(g m^–3^)	
π*_*l,u*_*	Osmotic potential of phloem @ loading (l) or unloading (u) zones	(MPa)	
Ψ_50,m_	Mesophyll water potential at which 50% of the mesophyll conductance is lost.	−2.0 MPa	Bartlett et al., 2016
Ψ_*m*_	Water potential- mesophyll	(MPa)	
Ψ_*s*_	Water potential- soil	−0.001 MPa (default); See Table 2 for drought	
Ψ_*x*_	Water potential - xylem	(MPa)	Vs Description: Phloem velocity in loading area, unit: (ms^−1^)

*If values are outputs of the model, then only units of the parameter are shown.*

### Plant Water Status and Gas Exchange

We separated the plant into two hydraulic elements, the mesophyll of a single leaf and the root-to-leaf xylem network, to capture the protective effect of vulnerability segmentation (Tyree and Ewers, 1991). The leaf accounts for at least 30% of whole-plant hydraulic resistance, with the mesophyll tissue accounting for about half of this resistance (Sack and Holbrook, 2006; Scoffoni et al., 2011). This generates a strong water potential gradient across the mesophyll that buffers the xylem and phloem against water stress. We adapted the water balance equations from Sperry et al. (1998) to calculate the mesophyll and xylem water volume at each timestep:


(2a) $$\frac{dW_{m}}{dt}=\int_{{\text{Ψ}}_{m}}^{{\text{Ψ}}_{x}}K_{m}d\text{Ψ}-g_{s}VPDLA$$

(2b) $$\frac{dW_{x}}{dt}=K_{x}\left({\text{Ψ}}_{\text{s}}-{\text{Ψ}}_{\text{x}}\right)-\int_{{\text{Ψ}}_{m}}^{{\text{Ψ}}_{x}}K_{m}d\text{Ψ}=0$$

where *W* is the water volume, Ψ is the water potential, and *K* is the hydraulic conductance of the mesophyll (subscript *m*) and xylem (subscript *x*). The *VPD* and Ψ_*s*_ are the environmental parameters, the vapor pressure deficit and soil water potential, respectively; *g*_*s*_ is the stomatal conductance, and *LA* is the area of a single leaf (see Table 1 for constant parameter values and Table 2 for the parameters varied across simulations).

**TABLE 2:** Parameters which varied during the simulations.

Symbol	Definition	Value
*R*_*p*_	Estimated structural resistance coefficient of the phloem such that R1 obtains > 0.6 MPa loading pressures under wet soil conditions (i.e., Ψ = −0.001 MPa)	2.4e^20^ m^–3^ (R1:; *Vitis vinifera*) 5.4e^20^ (R2:; *Liriodendron chinense*) 1.1e^21^ (R3:; *Ricinus communis*) 2.3e^21^ (R4:; *Robinia pseudoacacia*) 8.5e^21^ (R5:; *Gossypium barbadense*)
*V*_*maxL*_	Maximum rate of sucrose uptake (phloem loading)	1.58e^–7^ (Default, *N. tabacum*; Borstlap and Schuurmans, 2004) – see Table 3 for range of values used in Figure 7
*V*_*maxU*_	Maximum rate of sucrose export (phloem unloading)	=V_*maxL*_ × 1.2
Ψ_*s*_	Water potential of soil	−0.001 − (-)1 MPa

*For R_p_, sieve element resistances from stem tissue were based upon stem anatomical data from Liesche et al. (2017; see Eq. 1 for formulation).*

The mesophyll water volume is converted to a relative water content by dividing by the maximum water volume $\left(RWC_{m}=\frac{W_{m}}{V_{sat,m}}\right)$, and then to a water potential through the pressure-volume relationships:


(3) $${\text{Ψ}}_{m}=\left\{ \begin{array}{cc} \frac{\pi_{0}\left(1-a_{f}\right)}{RWC_{m}-a_{f}}-\pi_{o}-\varepsilon \left(1-\frac{1-a_{f}}{RWC_{m}-a_{f}}\right)\; & {\text{Ψ}}_{m}>\pi_{tlp}\\ \frac{\pi_{m}}{RWC_{m}}\; & {\text{Ψ}}_{m}\leq \pi_{tlp} \end{array}\right.$$

where π*_*o*_*, π*_*tlp*_*, *a*_*f*_, and ε are the leaf pressure-volume curve parameters of osmotic potential at full hydration turgor loss point, apoplasmic fraction, and cell wall modulus of elasticity, respectively (Bartlett et al., 2012). We considered the effects of mesophyll sugar content on water relations to be outside the scope of this study, and, thus, we assumed here that π_*o*_ was constant.

Water flow through the mesophyll was determined by integrating the mesophyll vulnerability curve:


(4) $$K_{m}=\int_{0}^{t}\frac{K_{max,m}}{1+{\text{e}}^{-\alpha \left({\text{Ψ}}_{m}-{\text{Ψ}}_{50,m}\right)}}$$

where *K*_*max,m*_ is the maximum hydraulic conductance of the mesophyll, normalized by leaf area, α is a shape parameter, and Ψ_50,*m*_ is the mesophyll water potential at which 50% of conductance is lost. To simplify these calculations, we used a sufficiently negative xylem Ψ_50_ value (−2 MPa) to assume *K*_*x*_ was constant and equal to the maximum xylem conductance (*K*_*max,x*_) over the range of xylem water potentials in these simulations.

We calculated *g*_*s*_ from the assumption that mesophyll water stress induced stomatal closure:


(5) $$g_{s}=\frac{g_{max}}{1+e^{-ags\left(\Psi_{m}-g_{s}\Psi_{50}\right)}}$$

where *g*_*max*_ is the maximum stomatal conductance, *ags* is the shape parameter for this relationship, and *g*_*s* 50_ is the mesophyll water potential inducing 50% stomatal closure. Photosynthesis (*A*_*net*_) was then calculated from *g*_*s*_ based upon an extrapolation of the original Farquhar equations (Farquhar et al., 1980; Bartlett et al., 2019).

At the beginning of each timestep, Ψ*_*m*_* was calculated from the change in mesophyll volume over the previous timestep (Eqs 2a, 3). Mesophyll water flow was then calculated by integrating the mesophyll vulnerability curve over water potential, bounded by the new Ψ*_*m*_* and the Ψ*_*x*_* from the previous timestep (Eqs 2, 3). Stomatal conductance (*g*_*s*_) was then calculated from the new Ψ*_*m*_* (Eq. 5), and Ψ*_*x*_* was updated from the new mesophyll water flow:


(6) $$\Psi_{x,t}=\Psi_{s}-\frac{\int_{{\text{Ψ}}_{m,t}}^{{\text{Ψ}}_{x},t-1}K_{m,t}d\text{Ψ}}{K_{max,x}}$$

where *t* indicates the current timestep and was used to determine the new xylem flow *K*_*max*,*x*_(Ψ_s_−Ψ_x_) (Eq. 2b). The new mesophyll flow and g_*s*_ values were then supplied to Eq. 2a to update the mesophyll volume for the next timestep. We ran the model at a 1s timestep over 12 h simulations to achieve steady-state solutions, which were reported as the model results.

### Phloem Transport

The mass of sucrose in the mesophyll (*S*_*m*_) was increased by photosynthesis and reduced by loading into the phloem companion cells via sucrose transporters:


(7) $$\frac{dS_{m}}{dt}=A_{net}LA-L_{sucL}LA$$

where *L*_*sucL*_ is the loading rate per unit leaf area. To simplify the model, we assumed all the sugar produced by photosynthesis is exported into the apoplasmic space surrounding the sieve element/companion cell complexes of the loading zone, and thus available for phloem loading. For loading (subscript *i* = *L*) or unloading (subscript *i* = *U*), *L*_*suc,i*_ was calculated from the Michalis-Menten formalism for enzyme kinetics:


(8a) $$L_{suc,i}=V_{max,i}\frac{C}{M_{S}+C}$$

(8b) $$C_{m}=\frac{S_{m}}{W_{m}}$$

where *V*_*max,i*_ is the maximum loading or unloading rate of the sucrose transporters, normalized by leaf area, and *M*_*S*_ is a shape parameter capturing transporter affinity for sucrose. *C* is either *C*_*m*_, the sucrose concentration in the mesophyll outside the loading zone, or *C*_*pU*_, the sucrose concentration in the unloading phloem zone. *W*_*m*_ is the mesophyll water volume (Eq. 2a), which changes the concentration of sucrose available for loading in Eq. 8b.

Loading increased the mass of sucrose in the phloem loading zone (*S*_*p,L*_), which was transported to the unloading zone:


(9) $$\frac{dS_{p,L}}{dt}=L_{SUCL}LA-E_{s}$$

where *E*_*s*_ is the mass flow rate of sucrose transport (g s^–1^). *E*_*s*_ was calculated from the volumetric flow rate of the phloem sap (*F*_*p*_; m^3^ s^–1^) and sucrose concentration in the loading zone (*C*_*p,L*_; mol m^–3^):


(10a) $$E_{s}=F_{p}C_{p,L}mm$$

(10b) $$C_{p,L}=\frac{S_{p,L}}{W_{p}}$$

where *mm* is the molar mass of sucrose and *W*_*p*_ is the maximum water volume in the loading zone. Thompson and Holbrook (2003a) found that accounting for *diurnal changes* in phloem volume did not substantially impact the flux of sucrose through the transport pipeline. Thus, we made the simplifying assumption that *W*_*p*_ was constant at the maximum loading zone phloem volume, so that *C*_*pL*_ only varied with the mass of sucrose.

The sucrose concentration determined the osmotic potential (π_*i*_) in the loading (*L*) or unloading zone (*U*):


(11) $$\pi_{i}=-RcTC_{p,i}$$

where *Rc* is the gas constant and *T* is the temperature within the phloem loading area. Following Thompson and Holbrook (2003b), we made the simplifying assumption that the water potential of the phloem loading zone equilibrates with the xylem water potential (Ψ_*x*_) when the phloem is at steady state. Further, we estimate that the anatomical dimensions, osmotic strength, and enhanced permeability due to aquaporins (Muries et al., 2013; Stanfield et al., 2017) of our loading zone has an RF (axial to radial resistivity) value >> 1 (see Thompson and Holbrook, 2003b), which equates to phloem in water potential equilibrium with the xylem. The turgor pressure in the loading or unloading zone (*P*_*i*_) was then determined from the water and osmotic potentials:


(12) $$P_{i}=\Psi_{x}-\pi_{i}$$

Noting that for determining the phloem pressure in the unloading zone, Ψ_*s*_ is used. The volumetric phloem flow rate F_*p*_ (m^3^ s^–1^) was calculated from the pressure difference between the loading (P_*L*_) and unloading (P_*U*_) zones and the hydraulic resistance of the phloem (*R*_*conduit*_):


(13) $$F_{p}=\frac{P_{L}-P_{U}}{R_{conduit}}$$

which increased with the phloem sucrose concentration due to viscosity (*v*) effects (see Appendix A), and integrated into a resistance formula:


(14) $$R_{conduit}=Rp\times v$$

where *Rp* is the resistance coefficient of the single phloem conduit, or sieve tube (see Table 2).

The velocity of sap flow (*V*_*s*_) was calculated by normalizing volumetric flow by conduit area:


(15) $$V_{s}=\frac{Fp}{\pi r^{2}}$$

where *r* is the radius of the conduit.

The same processes then take place when the sucrose reaches the unloading zone, where unloading is determined from:


(16) $$\frac{dS_{U}}{dt}=E_{s}-L_{SUCU}LA$$

We made the simplifying assumptions that the source and sink area (*LA*) and the phloem volume in the loading and unloading zones (*W*_*p*_) are equal.

Finally, we quantified sugar export (*S*_*tot*_) as the cumulative mass of sucrose unloaded over the simulation, as:


(17) $$S_{tot}=LA\int_{0}^{t}L_{SUCU}\left(t\right)dt$$

The model was implemented using MATLAB R2020a (9.8.0) (Mathworks, Inc., Natick, MA, United States). Access to the code may be obtained through doi: 10.5281/zenodo.5907490.

### Model Parameterizations to Test Hypotheses

We tested the impacts of water stress and phloem anatomy and transporter kinetics on phloem function by varying the (1) soil water potential (Ψ_*s*_), (2) phloem structural resistance (*Rp*), (3) phloem volume (*Wp*), (4) maximum sucrose loading rate (V_*maxL*_), and (5) the shape parameter for the relationship between loading rate (L_*sucL*_) and turgor (β) across simulations (see Table 2 for parameter values). We parameterized a range in soil water stress by varying Ψ_*s*_ from well-watered to droughted values (−0.001 to −1 MPa). We used the published sieve element dimensions for stems from 5 active loading species to produce reasonable estimates of *Rp* (Figure 1; see model assumption #7). Data for source phloem volume is scarce, so we initially estimated *Wp* by multiplying the mean sieve element area for *Populus tremula x alba* leaf (Carvalho et al., 2017) by the total vein length in a grapevine leaf, as a representative active loader (Pagano et al., 2016). However, this estimation only accounted for 0.002% of total volume in our hypothetical leaf, while the phloem has been estimated to account for up to 0.4% of leaf volume (Sjolund, 1997). Thus, we parameterized the model with phloem volumes ranging from 0.002 to 0.8% of leaf volume to capture a wide range of potential parameter space. We compared the cumulative sucrose export (S_*tot*_), phloem loading zone concentration (C_*l*_), pressure (P_*l*_), phloem viscosity (*v*) and velocity (V_*s*_) across simulations.

**FIGURE 3:**
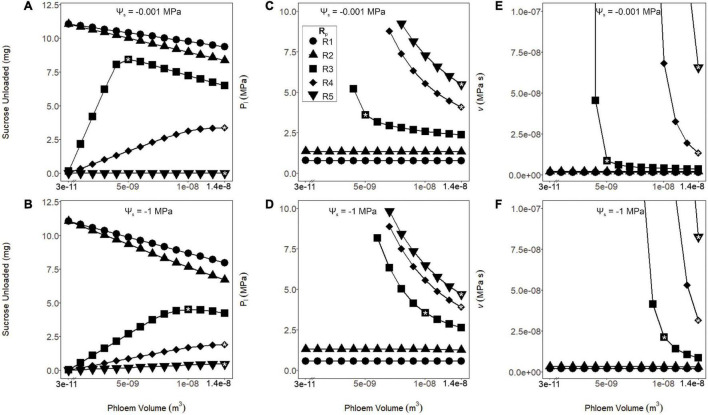
Sucrose unloaded, phloem pressure (P_*l*_) and viscosity (*v*) as a function of phloem volume and pathway resistance (R1–R5). These simulations were run under wet (Ψ_*s*_ = –0.001 MPa: **A,C,E**) or dry (Ψ_*s*_ = –1 MPa: **B,D,F**) soil conditions. **(A,B)** As phloem volume declined, sucrose export increased for the lower resistance pathways (R1 and R2). Export peaked at intermediate volumes and resistances (R3) and continued to increase at higher volumes and resistances (R4 and R5). This effect was intensified for the drier simulation. **(C,D)** Phloem pressure remained stable over all tested volumes for the lower resistance pathways (R1 and R2), while increased with lower volumes for the higher resistance pathways (R3–R5). For the higher resistance pathways and lower volumes, resulting pressures are not shown due to excessive values. **(E,F)** The phloem viscosity remained stable for lower resistance pathways (R1 and R2) over the range of volumes tested, while runaway viscosity began to occur at the lower phloem volumes for the higher resistance pathways (R3–R5). A drought scenario would induce the runaway viscosity effect at lower phloem volumes for these resistances. In subsequent analysis, volumes were chosen (white asterisks) for each resistance that maximized sucrose export while preventing the runaway viscosity effect over the range of Ψ_*s*_ tested: R1 and R2; 3.57 e^–11^ m^3^, R3; 1.04e^–8^ m^3^, and R4 and R5; 1.37e^–8^ m^3^.

Phloem resistance and volume had strong interactive effects on sugar export, with small volumes exacerbating viscosity limitations for high resistances (see “Results,” Figure 3). Thus, we were concerned that incorrect assumptions about these parameter combinations could overestimate viscosity limitations under drought. We used our first simulations to identify the phloem volumes that (1) maximized sugar export with (2) viable phloem pressures under drought (Ψ_*s*_ = −1 MPa) for each *Rp* value. We used these values to parameterize the rest of the simulations, to evaluate the impacts of transporter kinetics on viscosity limitation under the most favorable anatomical parameterizations.

Next, we parameterized variation in maximum loading rate (V_*maxL*_) by compiling published values for low (*Arabidopsis*), intermediate (tobacco), and high (wheat) rates (Weise et al., 2000; Borstlap and Schuurmans, 2004; Liesche and Schulz, 2013). We used the intermediate value from tobacco as our default unless otherwise noted (Table 3). We also tested whether regulating loading rates in response to pressure would protect the phloem from viscosity limitations, by reducing loading before reaching excessive concentrations and pressures. We used an exponential decay function to reduce the loading rate as a function of loading zone pressure:


(18) $$L_{suc,L}=V_{max,L}e^{-\beta Pl}\frac{C}{M_{S}+C}$$

and increased the shape parameter (β) from 0 to 10 to test the impacts of loading downregulation. We first varied β and V_*maxL*_ independently, and then together, to identify the combinations that (1) maximized sucrose export and (2) obtained viable loading zone pressures (see model assumptions #8) for different resistances (R2 and R4) and soil moisture scenarios (Ψ_*s*_ = −0.001 and −1 MPa).

**TABLE 3:** Data on available apoplasmic active loader maximum loading rate (V_*max*_) and phloem anatomical characters to calculate *R*_*p*_.

Species	Maximum loading rate (mol m^–2^ s^–1^) (V_*max*_)	Rp (for one sieve element)	References
Nicotiana tabacum	1.6e^–7^ (1), 3.3e^–7^ (2)	9.86E+17 (3) (petiole)	Borstlap and Schuurmans, 2004 (1); Cataldo, 1974 (2); Thompson and Wolniack, 2008 (3)
Beta vulgaris	1.6e^–7^ (1), 8.2e^–7^ (2)	–	Fondy and Geiger, 1977 (1); Sovonick et al., 1974 (2)
Vicia faba	1.60e^–7^	–	Delrot and Bonnemain, 1980
Trticum aestivum	2.30e^–6^	–	Kuo et al., 1974
Arabidopsis thaliana	1.30e^–8^ (1)	2.03E+19 (2) (stem)	Weise et al., 2000 (1); Thompson and Wolniack, 2008 (2)
Solanum tuberosum	1.10e^–8^	–	Weise et al., 2000
Pisum sativum	2.90e^–6^		Wimmers and Turgeon, 1991

## Results

### Coordination Between Phloem Anatomy Traits Strongly Influenced Phloem Vulnerability to Viscosity Limitation

**FIGURE 4:**
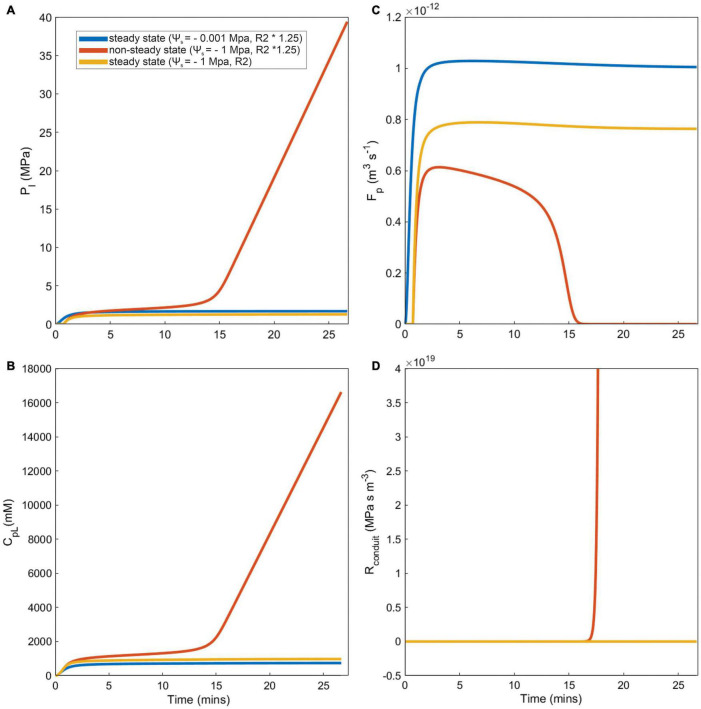
Examples of steady state, and non-steady state simulations (caused by runaway viscosity) for different model outputs, over a subset of total simulation run time. Three scenarios represented by each line color are presented for differing soil water conditions (Ψ_*s*_) and phloem structural resistance (R2): First, a steady state wet and higher phloem pathway resistance simulation (Ψ_*s*_ = –0.001, R2 × 1.25, blue lines). Second, a non-steady dry and higher resistance phloem pathway simulation (Ψ_*s*_ = –1, R2 × 1.25, red lines). Finally, a steady state dry and lower resistance pathway simulation (Ψ_*s*_ = –1, R2, yellow lines). Each panel represents **(A)** Phloem pressure in the loading area (P_*l*_), **(B)** concentration of sucrose in phloem loading area (C_*pL*_), **(C)** phloem sap flow (F_*p*_), and **(D)** total resistance of the phloem sieve tube conduit in the loading area, which considers fluid viscosity and phloem structural resistance (R_*conduit*_).

The importance of viscosity limitations on sugar transport depended strongly on phloem anatomy (Figure 4). Simulations either reached stable, equilibrium sucrose concentrations over the course of the 12-h model runs (i.e., steady-state), or failed to converge on a stable concentration due to excessive viscosity (i.e., non-steady-state) under high water stress and/or sieve tube structural resistances and small phloem volumes. For example, a simulation with moderate structural resistance (R2) reached steady-state and achieved a stable loading zone concentration, pressure, total resistance, and flow rate under dry conditions (ψ_*s*_ = −0.001 and −1 MPa) (Figure 4, yellow lines). By contrast, a simulation with ∼25% greater structural resistance still reached steady-state in wet soil (Figure 4, blue lines), but failed to equilibrate under water stress and phloem concentration and pressure increased without limit (Figures 4A,B, red lines). In these non-steady-state simulations, phloem viscosity and total resistance remained too high for the loading zone pressure to overcome (Figure 4C, red line), even as concentrations increased, preventing phloem flow (Figure 4D, red line). In these scenarios, sugar export only proceeded for a small fraction of the 12-h simulation.

Coordinating phloem volume with pathway resistance alleviated runaway viscosity and avoided non-steady-state transport failure. There is little data available to parameterize phloem volume and resistance in leaves for active loading species, so we simply varied these parameters widely (i.e., by three orders of magnitude for phloem volume; see Methods) to evaluate the impacts on sucrose export (Figures 3A,B), phloem pressure (P_*l*_; Figures 3C,D) and viscosity (*v*; Figures 3E,F). In the lower resistance pathways (R1 and R2), all phloem volumes allowed sucrose export to proceed without runaway viscosity, while the smallest volumes produced the greatest sucrose output, under both wet and dry conditions (Ψ_*s*_ = −0.001 and −1 MPa). However, for the intermediate (R3) and high (R4 and R5) phloem resistances, phloem pressure and viscosity reached intractable values at lower volumes; this effect was exacerbated under drier soils. Overall, larger phloem volumes were optimal for sucrose export in more resistive pathways and drier conditions. We also used the volumes that optimized export under drought (white asterisks) to parameterize volume for each resistance in the subsequent simulations, to avoid overestimating the importance of viscosity limitations by making incorrect assumptions about phloem anatomy (i.e*., W*_*p*_ = 3.57e^–11^ m^3^ for R1 and R2, 1.04e^–8^ m^3^ for R3, and 1.37e^–8^ m^3^ for R4 and R5; see “Materials and Methods” and Table 2).

### Sucrose Export Was Independent of Phloem Resistance and Xylem Water Status Until These Variables Reached Thresholds for Viscosity Limitation

**FIGURE 5:**
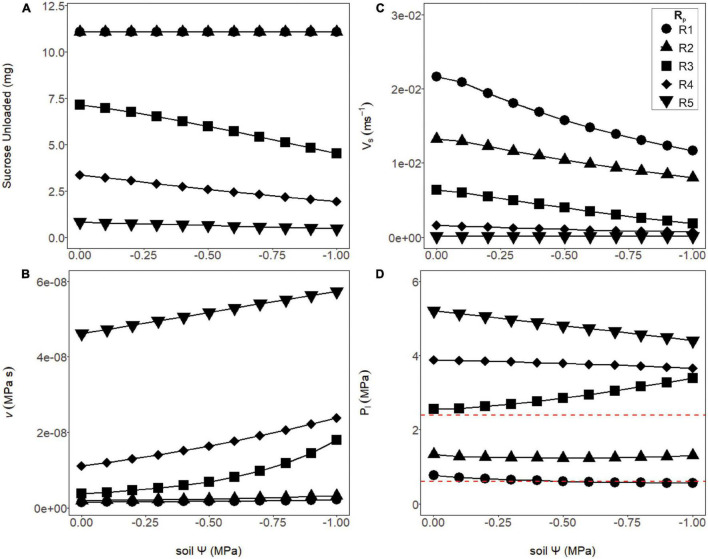
Phloem output parameters modeled over varying soil water potentials and phloem resistance coefficients. **(A)** The amount of sucrose unloaded was consistent across soil water potentials for the first two structural resistance (*R*_*p*_) values (R1 and R2), while R3–R5 exported a substantially lower amount. **(B)** The viscosity of the phloem sap increased as *R*_*p*_ increased, and the soil became drier. Note the intermediate resistance began to increase exponentially, as a sign that this resistance was approaching viscosity limitation. **(C)** The velocity of phloem transport declined with drier soil, as well as increasing (*R*_*p*_). **(D)** Phloem pressures declined slightly under drier soils for the lower or higher resistance phloem (R1, R2 and R4, R5) but began increasing with drier soil at the intermediate resistance (R3) due to this resistance approaching viscosity limitation. Red dashed lines signify phloem pressure potentials measured empirically in past studies (0.6–2.4 MPa).

Sugar export was constant over the soil water potentials tested for structural resistances below thresholds for viscosity limitation (Figure 5A, R1 and R2), and declined above these thresholds (Figure 5A, R3–R5). Below these thresholds, loading zone concentration and turgor increased until flow reached a steady-state equilibrium (see Figure 4), where the mass of sucrose imported into the phloem equaled the mass exported. Here, we assumed transport was not constrained by unloading and that all sucrose that entered the unloading area was immediately exported to sinks (i.e., V_*maxU*_ >> V_*maxL*_; see “Materials and Methods”: assumption #2). Thus, sucrose export in these lower resistance simulations (R1 and R2) were independent of water stress and phloem pressure. Drier conditions increased sap viscosity and reduced phloem velocity by changing the total resistance and source to sink pressure gradient (Figures 5B,C). For the least resistant phloem (Figure 5D, R1), phloem pressure slightly decreased with soil water potential, because the xylem water potential was decreasing at a greater rate than phloem osmotic potential (e.g., Supplementary Figure 3, green line).

In contrast, the simulations with the most resistant sieve tubes (R3–R5) not only declined in their overall sucrose output but varied in their response to the soil dry-down (Figure 5A). The decline in sucrose output was attributed to a viscosity limitation, as the higher resistance scenarios had both higher overall viscosity, and increased viscosity under higher drought stress (Figure 5B). As with the lower resistance pathways, velocity declined with soil water potential, but did so more rapidly (Figure 5C). Finally, phloem pressures in the higher resistance pathways were well above empirically measured values of 0.6–2.4 MPa (Figure 5D, red dashed lines). Of note was the intermediate resistance (R3) which showed increased phloem pressure with declining soil water, as a result of phloem osmotic potential increasing faster than xylem water potential (Supplementary Figure 3, red line).

### Maximizing Export While Maintaining Viable Phloem Pressure Using Pressure Regulated Loading

**FIGURE 6:**
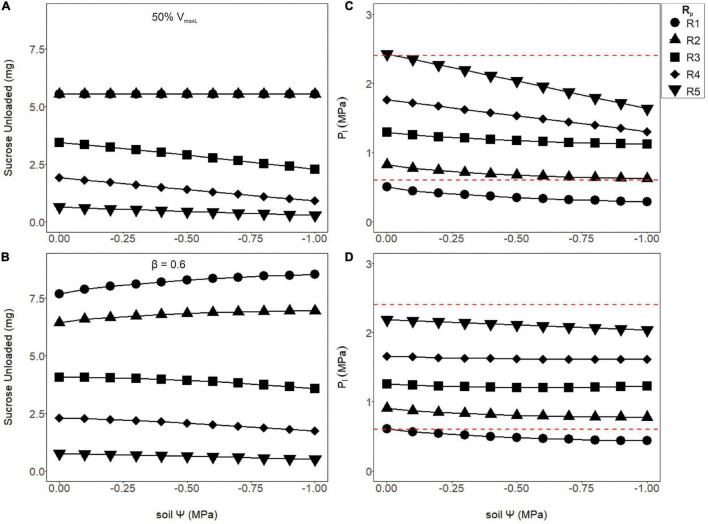
The amount of sucrose unloaded or phloem pressure in the loading zone (P_1_) as a function of soil water potential (Ψ_*s*_) for two differing loading rate scenarios: a 50% reduction in V_*maxL*_ **(A,C)** or a pressure regulated reduction in the default VmaxL, with a downregulation strength of β = 0.6 (**B,D**; see Eq. 18). The first scenario **(A,C)** shows the amount of sucrose unloaded and P_1_ for a 50% reduction in V_*maxL*_. Output remained stable over the drought for R1 and R2 and began to decline with soil water for R3–R5. Meanwhile, phloem pressure began to fall with drier soils, with most *R*_*p*_ values falling within the empirical pressure window, except for the lowest pathway resistance which was under-pressured. **(B,D)** Using pressure downregulated loading, sucrose output slightly improved as the soil dried for lower *R*_*p*_ values (R1 and R2) but started to decline with higher values (R3–R5). Downregulation of loading allowed most of the tested resistances (R2–R5) to have phloem pressures within empirical bounds. Red dashed lines signify phloem pressure potentials recorded previously empirically (0.6–2.4 MPa).

Although increasing phloem volume helped alleviate runaway viscosity, higher resistance pathways where still under the effects of viscosity limitation, which caused phloem pressures to be unrealistic. Thus, we hypothesized maximum loading rates to be modulated according to these anatomical constraints, by either downregulating V_*maxL*_ constitutively (Figures 6A,C) or inducibly as a function of pressure (Figures 6C,D). Reducing the maximum loading rate by half (V_*maxL*_) reduced sucrose output to sinks (Figure 6A) in comparison to the non-regulated loading, but lowered phloem pressures to reasonable bounds (Figure 6C, red dashed lines). However, using Eq. 18 to downregulate phloem loading as a function of pressure improved overall sucrose output over simply reducing V_*maxL*_ while maintaining viable phloem pressures over most soil water conditions (Figures 6B,D). Comparing wet soils (Ψ_*s*_ = −0.001MPa) between unregulated loading (Figure 5A) to pressure regulated loading (Figure 6B), sucrose output dropped by 30.6, 27.8, and 2.36% for R1, R3, and R5 resistances, respectively (β = 0.6). However, under dry soils (Ψ_*s*_ = −0.001 MPa), sucrose output dropped by 22.9 and 8.5% for R1 and R3, respectively, but for the highest resistance (R5), improved by 3.2%. This led to the hypothesis that pressure regulated loading may be optimized according to both sieve tube anatomical traits (e.g., *Rp*), environmental conditions (e.g., Ψ_*s*_) or maximum loading rates (e.g., V_*maxL*_).

### Maximizing Sucrose Export to Sinks Varies With Pressure-Regulated Maximum Loading Rate and Sieve Tube Resistance

**FIGURE 7:**
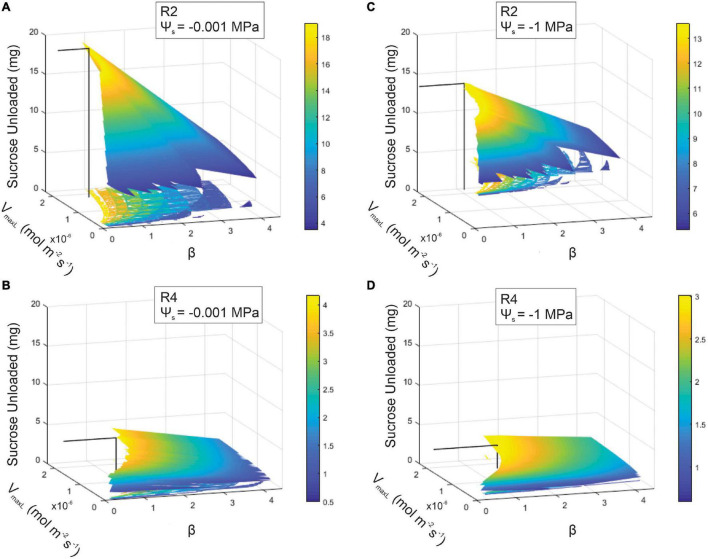
Sucrose output as a function of loading downregulation (β) and the maximum rate of sucrose loading (V_*maxL*_) over differing soil water potentials (Ψ_*s*_ = –0.001 or –1 MPa) or phloem structural resistances (*Rp*: R2 or R4). A range of V_*maxL*_ values were selected from *Arabidopsis* (1.30e^–8^ mol m^–2^ s^–1^) to wheat (2.3e^–6^ mol m^–2^ s^–1^). Sucrose output for: **(A)** R2 and wet soils (maximum sucrose output = 19.07 mg), **(B)** R4 and wet soils (maximum sucrose output = 4.18 mg), **(C)** R2 and dry soils (maximum sucrose output = 13.6 mg), and **(D)** R4 and dry soils (maximum sucrose output = 2.98 mg). The solid black line within each 3D space specifies the maximum sucrose output at a particular V_*maxL*_ and β.

We next sought to determine how the transporter kinetics that maximize sucrose export while maintaining viable phloem pressures depend on pathway resistance and soil moisture. We first identified the range of viable β values for each resistance and soil moisture scenario (i.e., Ψ_*s*_ = −0.001 and −1 MPa), assuming a constant V_*maxL*_ (i.e., for *N. tabacum*) (Supplementary Figure 4). We then varied V_*maxL*_ from 1.30e^–8^ (i.e., *Arabidopsis*) to 2.30e^–6^ mol m^–2^ s^–1^ (i.e., wheat) to identify the combinations of V_*maxL*_ and β that maximized export for each scenario (Figure 7).

Coordination between loading downregulation and the maximum loading rate strongly benefitted sucrose export (Figure 7). This simulation showed sugar export for the β and V_*maxL*_ combinations that produced viable phloem pressures (i.e., 0.6–2.4 MPa). For low-resistance phloem (R2) under wet conditions, sucrose export was maximized at 19.07 mg by V_*maxL*_ = 1.79e^–6^ mol m^–2^ s^–1^ and β = 0.79 (Figure 7A). Drought reduced the maximum output at this *Rp* to 13.6 mg (i.e., a 29% reduction) (Figure 7C), which occurred at a higher V_*maxL*_ (2.22e^–6^ mol m^–2^ s^–1^) with stronger pressure-coupled downregulation (β = 1.14); in comparison to default V_*maxL*_, the expanded V_*maxL*_ improved export by 50 and 28% for the wet and dry conditions, respectively, at this resistance.

Increasing resistance (R4) in wet conditions substantially reduced maximum output to only 4.18 mg (Figure 7B); this corresponded to a lower V_*maxL*_ and stronger downregulation than for R2 (V_*maxL*_ = 1.68e^–6^ mol m^–2^ s^–1^ and a β = 1.33). Water stress reduced maximum output to 2.98 mg (a 29% reduction) (Figure 7D) and this corresponded to the strongest downregulation across scenarios (V_*maxL*_ = 1.72e^–6^ mol m^–2^ s^–1^ and β = 1.61); this improved sucrose output by 27 and 33% over default V_*maxL*_ values for wet and dry conditions, respectively. Overall, when comparing the impacts of pressure regulated loading (Figure 7) vs. non-regulated loading (Figure 5), we found that non-water stressed, low resistant pathways and water stressed, high resistant pathways benefit the most from a pressure regulated loading mechanism (Table 4).

**TABLE 4:** Comparing the maximum sucrose output of the unregulated phloem loading scenario (Figure 5), to the pressure regulated loading scenario (data found in Figure 7).

Phloem resistance (*R*_*p*_)	No regulation sucrose output (mg)	Pressure regulation sucrose output (mg)	% Change
**Ψ_*s*_ = −0.001 MPa**			
R1	11.06	41.02	271
R2	11.05	19.07	73
R3	7.13	9.22	29
R4	3.35	4.18	25
R5	0.81	1.06	30
**Ψ_*s*_ = −1 MPa**			
R1	11.05	27.75	151
R2	11.05	13.6	23
R3	4.5	6.18	37
R4	1.91	2.98	56
R5	0.48	0.81	70

## Discussion

This study is the first to show that pressure-regulated loading is a potential strategy to circumvent viscosity limitations and improve the efficiency of sugar translocation, especially under drought and for highly resistive phloem pathways. Our findings also suggest sucrose loading proteins and phloem architecture (e.g., sieve tube anatomy) are highly coordinated. We discuss the potential molecular mechanisms driving pressure-adjusted loading during drought, the interacting effects between anatomy and viscosity limitations, and the potential for this model to inform future genetic transformation studies to increase crop yields.

### Plants Benefit From Loading Regulation: A Molecular Regulation Perspective

Our study provides theoretical support for our hypothesis that loading regulation is adaptive for phloem function. Pressure-regulated loading prevented the excessive buildup of sugars that causes viscosity limitations, reducing the impacts of water stress on sugar transport for highly resistive phloem (Figures 6, 7). Regulated loading also benefitted less resistive pathways by allowing for a higher V_*maxL*_ and, thus, greater sugar export, than in the absence of regulation (Table 4). These model findings are bolstered by empirical evidence supporting pressure-regulated behavior and the discovery of molecular mechanisms that could dynamically adjust sucrose loading during drought.

Previous experiments have shown that loading rates may be adjusted as a function of turgor and/or sucrose concentration during osmotic stress. For example, exposing tissues to membrane-impermeable sugars (i.e., sorbitol or mannitol) reduces the apoplasmic water potential outside the phloem, drawing out water and reducing phloem turgor (Smith and Milburn, 1980; Patrick, 1994; Bell and Leigh, 1996). These treatments showed that a decline in phloem pressure upregulated sucrose loading in castor bean leaf discs (Smith and Milburn, 1980) and sucrose unloading in bean seed coats (Patrick, 1994). Conversely, reducing external mannitol concentrations increased phloem turgor and decreased unloading rates in beet root disks (Bell and Leigh, 1996). Supporting loading downregulation, isolated vesicles from beet leaf disks exposed to increasing sucrose concentrations showed declines in loading rate in a concentration-dependent manner (Chiou and Bush, 1998). This downregulation also corresponded to a reduction in sucrose symporter transcript abundance with increasing sucrose concentrations, causing maximum loading rate (V_*max*_) to decline by up to 74% compared to controls which were not subjected to increased sucrose concentrations.

The empirical and modeling results suggest that plants would benefit from the ability to exhibit a range of loading rates, while the directionality of loading responses would depend on internal phloem conditions (e.g., anatomy or viscosity). For example, the model predicted that more resistive phloem would require stronger downregulation to optimize sugar transport under drought than less resistive pathways (Figures 7B,D; R4 vs. Figures 7A,C; R2). Weaker downregulation would allow excessive sucrose concentrations to build in the phloem, causing pressure and viscosity to rise and flow rates and export to decline (Figure 6B; R3–R5). Thus, upregulation could be beneficial during drought if resistance is below thresholds for viscosity limitation, allowing greater osmotic strength to compensate for the lowered xylem water potentials.

Changes in the regulation of sucrose transporter activity may be the primary cause of loading up- or downregulation and several mechanisms could allow these changes to occur in response to drought. Alteration in phloem turgor have been hypothesized to trigger a hormonal signaling cascade that alters transporter expression or post-translational modification (Patrick et al., 2001). This has been evidenced by water stress upregulating most (but not all) sucrose transporter subfamilies involved in phloem loading in some species (Medici et al., 2014; Xu et al., 2018). For example, *Arabidopsis*, soybean, barley, rice, wheat, and maize saw an overall upregulation of phloem loading SUT expression, but potato and tomato did not. However, phloem exudates from these studies indicate that sucrose concentrations decreased under water stress for all species, which contradicts the hypothesis that SUT transcript abundance alone drives differential loading rates but is consistent with our model results for differential control of V_*maxL*_ and pressure down-regulation of loading (Figure 7). Alternatively, post-translational modification of transporter proteins may regulate the rate of sugar movement past the phloem membrane (reviewed by Liesche et al., 2011a). For instance, ubiquitination of these proteins increases their degradation rate in the plasma membrane, while phosphorylation increases their affinity for sucrose in plants exposed to differing light (Xu et al., 2020) or drought (Ma et al., 2019). Interestingly, in high light conditions, photosynthesis and SUC2 phosphorylation were significantly increased, while SUC2 transcript remained stable (Xu et al., 2020). This implies that SUC/SUT transporter regulation is complex and under multiple controls. Future experiments may incorporate a variety of progressively intensified abiotic challenges (e.g., high light intensity, drought) and track both expression levels and post-translational modifications of SUTs/SUCs; this would help determine if thresholds of sucrose transporter upregulation exist, beyond which downregulation occurs to prevent viscosity limitation.

### Loading Regulation to Prevent Viscosity Limitation May Extend to the Pre-phloem Pathway

The current work focuses on the role of regulating loading proteins at the companion cell/sieve element interface. However, there is emerging evidence that phloem loading may be controlled in the pre-phloem pathway (Liesche, 2017), which would expand the applicability of studying viscosity limitations past strict apoplasmic loaders; for example, passive loaders or species with alternating loading types (i.e., English Oak; Liesche, 2017). Understanding how loading regulation varies between species may also help to elucidate carbon allocation patterns in response to stress (Savage et al., 2016).

For example, poplar (*Populus trichocarpa*) shows regulation of sucrose in the pre-phloem pathway through differential expression of tonoplast sucrose transporters in mesophyll cells (Payyavula et al., 2011). Under drought stress, these transporters were downregulated, stem growth reduced, and leaf sugar accumulation increased (Frost et al., 2012), signaling that phloem export from the leaf was diminished (Liesche, 2017). It could be hypothesized that if the *Populus* phloem was on the verge of viscosity limitation, sucrose needed to be withheld from entering the phloem loading pathway by being sequestered in vacuoles to prevent viscosity build-ups. Alternatively, other species such as beech (*Fagus sylvatica*) did not show reduced carbon export from the leaf during drought, but instead saw a reduction in photosynthesis (Hommel et al., 2016; Liesche, 2017). Beech has been identified as a passive symplasmic loader (Rennie and Turgeon, 2009), which may make it more reliant on adjustments of photosynthesis to control proper sucrose gradients in the phloem. Future work will need to identify sucrose transporter proteins in both the pre-phloem and phloem pathways to understand what role loading regulation plays in supporting efficient phloem transport across multiple plant taxa with differing loading strategies.

### Linking Anatomical Traits With Phloem Export to Sinks

Pressure-based loading improved sucrose export to sinks over non-regulated scenarios by up to 3.7x, while the lowest resistance pathway (R1) improved loading over the highest (R5) by 38.7x (Table 4). This might imply that plants with higher growth rates correlate to a lower resistance phloem pathway. However, a recent meta-analysis on phloem anatomical traits did not find a significant trend between growth rates and sieve tube resistance (Liesche et al., 2017) but did find increased variability in taxonomic groups that actively load. Potentially, loading regulation allows for higher pathway resistances to achieve similar levels of export to sinks in comparison to non-regulated pathways, which would make anatomical characters less constrained.

Another interesting interaction in phloem anatomy that was highlighted here was the relationship between phloem volume and resistance. In our simulations, export was maximized at phloem volumes that were small enough for rapid loading, but large enough to avoid viscosity limitations (Figure 3). Further, optimal volumes were larger for more resistant pathways. The relationship between phloem volume and resistance would depend on the underlying traits; for example, doubling conduit radius would increase phloem volume four-fold and reduce resistance 16-fold, while doubling the number of parallel conduits per unit area would decrease resistance and increase volume linearly (Hölttä et al., 2009; Jensen, 2019). This flexibility suggests it is highly plausible for natural or artificial selection to achieve optimal coordination between resistance and volume, though more work is needed to determine whether the inverse relationships between volume and resistance would prevent highly resistive pathways from achieving optimal volumes.

### Using Loading Regulation Mechanisms to Improve Genetic Engineering Outcomes

Previous studies have used genetic engineering to upregulate the expression of phloem loading sucrose transporters in pea (*Pisum sativum*; Lu et al., 2020), *Arabidopsis* (Dasgupta et al., 2014), potato (*Solanum tuberosum*, Leggewie et al., 2003), and rice (*Oryza sativa;* Wang et al., 2015). Although these transformations increased loading rates (Lu et al., 2020), the impacts on growth and viability varied by species. One successful example from pea plants saw the upregulation of *Ps*Sut1 which increased sucrose concentrations in the phloem exudate and significantly increased biomass and yield (Lu et al., 2020). The authors hypothesized that upregulating SUT1 enhanced both loading and unloading in the developing seeds, which was key to making this transformation successful (Lu et al., 2020). Similarly, we found that reducing sink limitations would minimize the buildup of sucrose that would encourage viscosity limitation (Supplementary Figure 1). Our study also suggests that lowering phloem pathway resistance in coordination with increasing maximum loading rate would lower pressure induced downregulation and increase sucrose output substantially (Figure 7). This could be achieved by screening potential lines of SUT transformation for sieve tube anatomical characteristics that lower resistance, such as sieve element length, diameter, and sieve plate porosity (Stanfield et al., 2019). While sieve tube anatomy may be a difficult genetic target, reducing plant height (dwarfing) could be a simple method to reduce pathlength resistance (e.g., Qiao and Zhao, 2011) to determine the interacting effects of SUT upregulation, lowered pathway resistance and yield. Further, elucidating the mechanisms that generate turgor-dependent signaling cascades that modify sucrose transporter expression (as suggested by Patrick et al., 2001) could allow genetic engineering approaches to optimally target maximum loading rates.

## Conclusion

We present a model that highlights the interactive effects of regulated phloem loading, phloem architecture, and drought on the total export to sink tissue. We find that phloem pathway resistance and maximum sucrose loading rate (V_*max*_) are coordinated and that higher resistance phloem pathways may experience viscosity limitations which result in diminished sucrose export to sinks. After coordinating phloem structural resistance with phloem volume, we hypothesized that the loading rate requires pressure-induced regulation to ease viscosity limits and maximize phloem export. Using pressure regulated loading, we found that phloem transport could be made more efficient across all phloem pathway resistances, and that this mechanism buffered against the effects of moderate drought stress. We suggest future studies that use genetic engineering tools to upregulate the abundance of phloem loading SUTs/SUCs integrate phloem architecture and regulatory pathways that control transporter expression in response to phloem water status. Studying the interactive effects of these traits has the potential to provide pathways to increase crop yield, and to elucidate the drivers of plant growth and mortality responses to climate change.

## Data Availability Statement

The datasets presented in this study can be found in online repositories. The names of the repository/repositories and accession number(s) can be found below: doi: 10.5281/zenodo.5907490.

## Author Contributions

RS constructed the figures and performed the literature review. RS and MB constructed the model, analyzed the results, wrote the manuscript, contributed to the article, and approved the submitted version.

## Conflict of Interest

The authors declare that the research was conducted in the absence of any commercial or financial relationships that could be construed as a potential conflict of interest.

## Publisher’s Note

All claims expressed in this article are solely those of the authors and do not necessarily represent those of their affiliated organizations, or those of the publisher, the editors and the reviewers. Any product that may be evaluated in this article, or claim that may be made by its manufacturer, is not guaranteed or endorsed by the publisher.

## Appendix A

The fluid viscosity of phloem sap was calculated using:

(1A)
$$v=v_{w}e^{\left(0.032Sf-{\left(0.012Sf\right)}^{2}+{\left(0.023Sf\right)}^{3}\right)}$$

(2A)
$$Sf=\frac{CpL\times mm}{\rho }$$

(3A)
$$\rho =\frac{CpL}{1000}0.1256+1.0019$$

where ρ and *v* are the density and viscosity of the phloem sap, respectively. These equations follow Jensen et al. (2013), where *v* is calculated from *v*_*w*_, the viscosity of pure water at 20°C, and *Sf*, the mass fraction of sucrose in the phloem (w/w), and ρ is calculated as a linear function of phloem sucrose concentration.
