# Supplementary material for: Coordination Between Phloem Loading and Structure Maintains Carbon Transport Under Drought
Source: Front Plant Sci. 2022 Feb 17;13:787837. doi: 10.3389/fpls.2022.787837 (PMC8891486; doi:10.3389/fpls.2022.787837)
Supplement: Supplementary file 1 [file Presentation_1.pptx]

## Slide 1
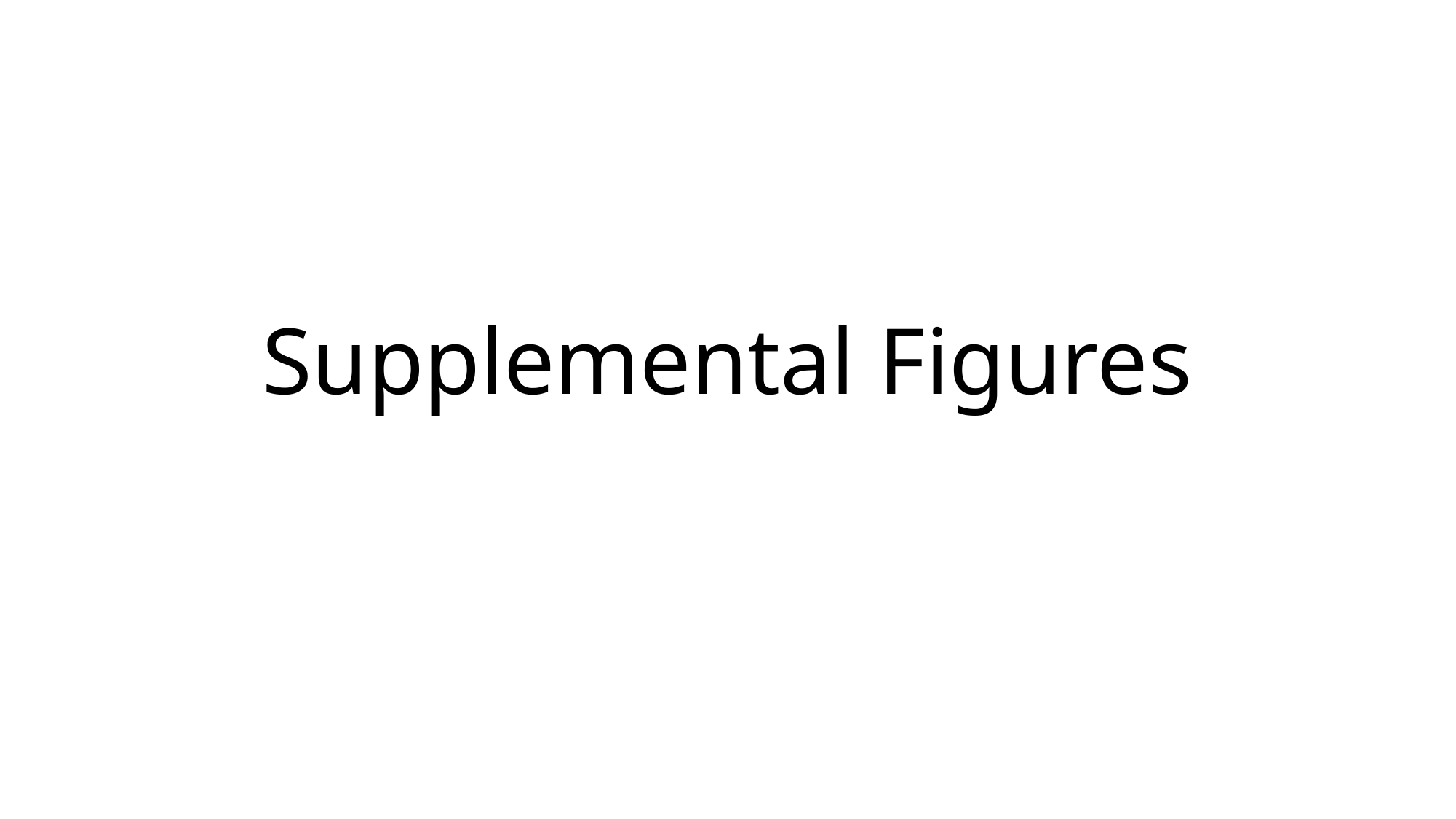

# Supplemental Figures

## Slide 2
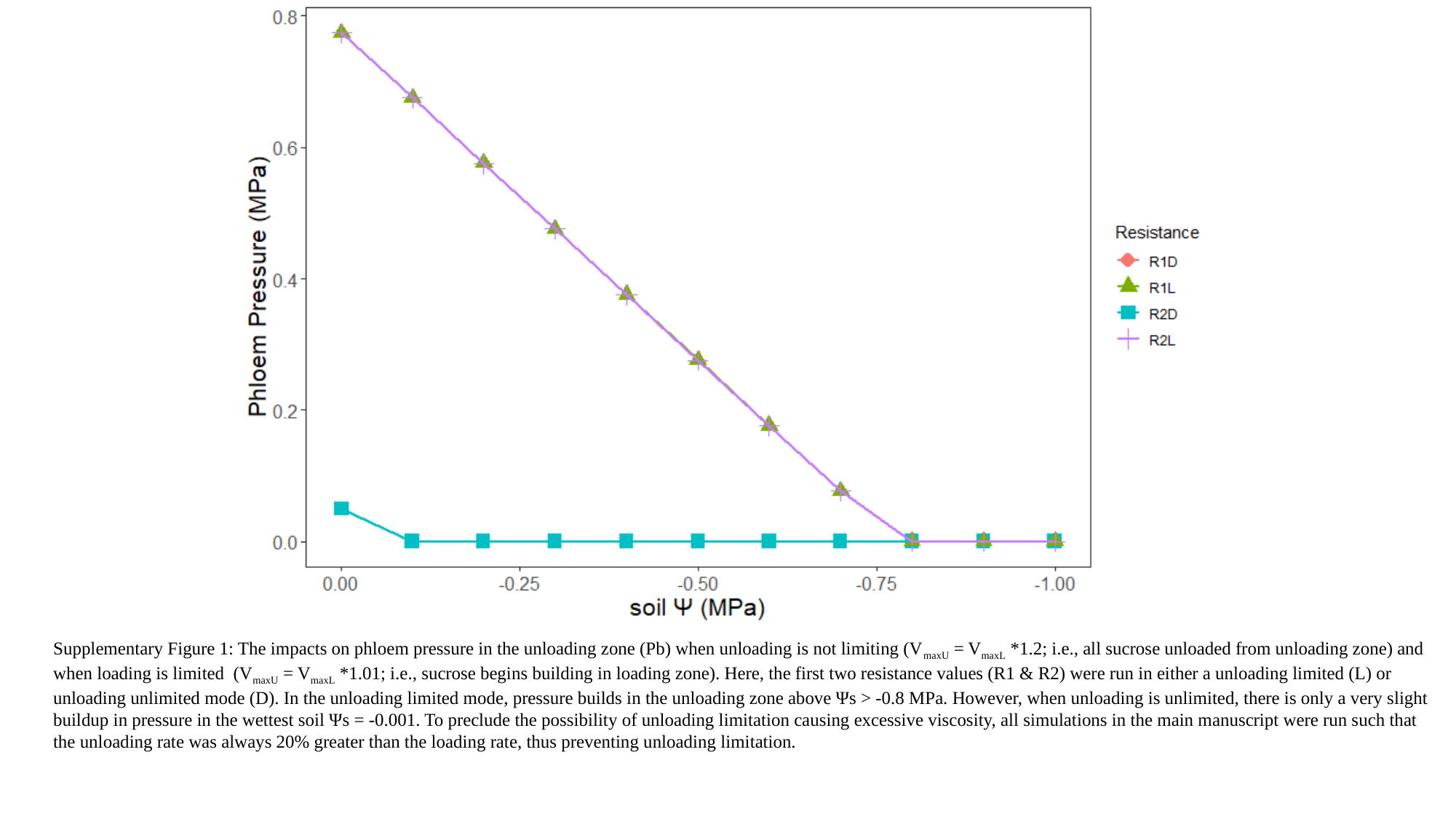

Supplementary Figure 1: The impacts on phloem pressure in the unloading zone (Pb) when unloading is not limiting (VmaxU = VmaxL *1.2; i.e., all sucrose unloaded from unloading zone) and when loading is limited (VmaxU = VmaxL *1.01; i.e., sucrose begins building in loading zone). Here, the first two resistance values (R1 & R2) were run in either a unloading limited (L) or unloading unlimited mode (D). In the unloading limited mode, pressure builds in the unloading zone above Ψs > -0.8 MPa. However, when unloading is unlimited, there is only a very slight buildup in pressure in the wettest soil Ψs = -0.001. To preclude the possibility of unloading limitation causing excessive viscosity, all simulations in the main manuscript were run such that the unloading rate was always 20% greater than the loading rate, thus preventing unloading limitation.

## Slide 3
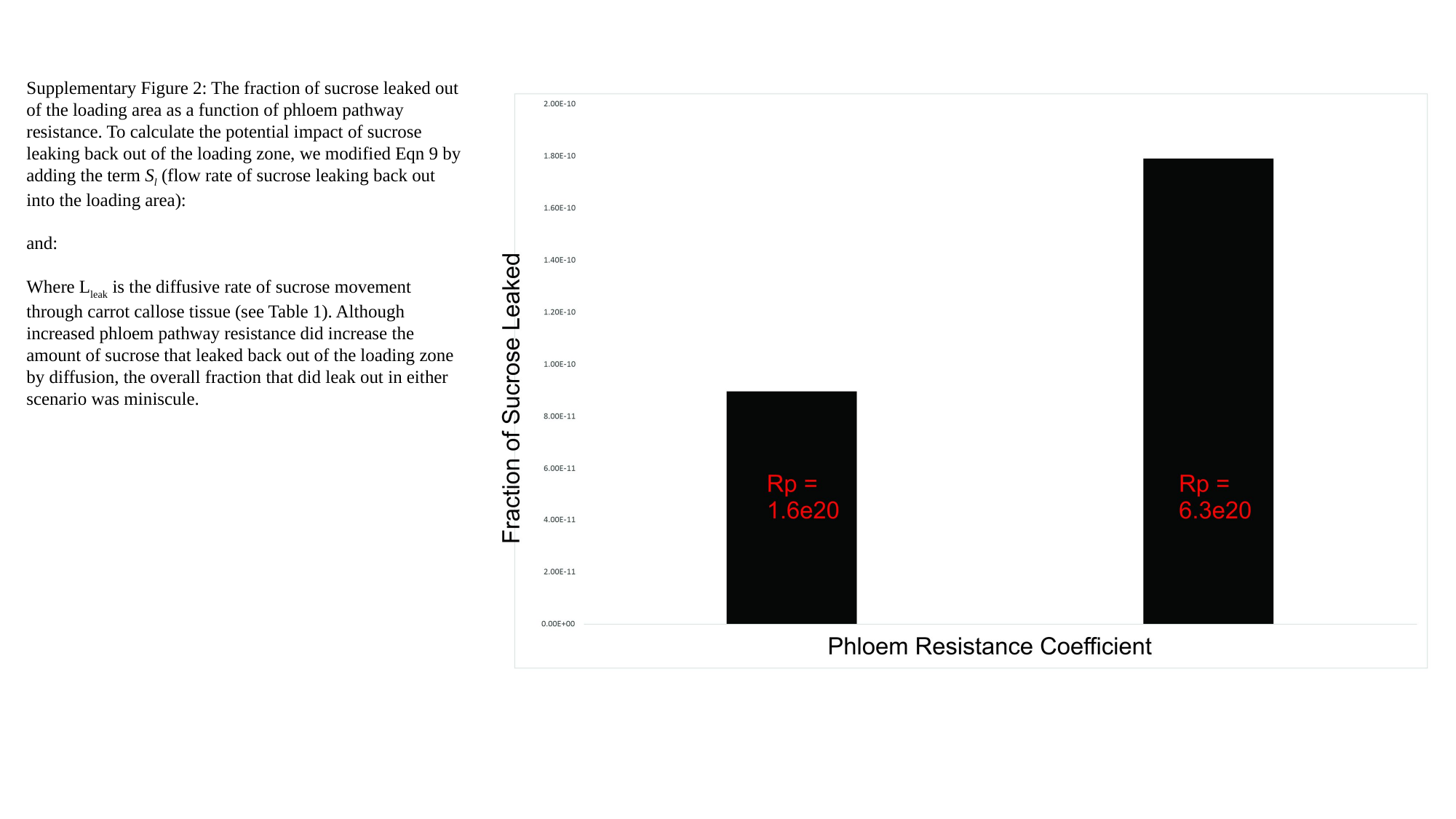

## Slide 4
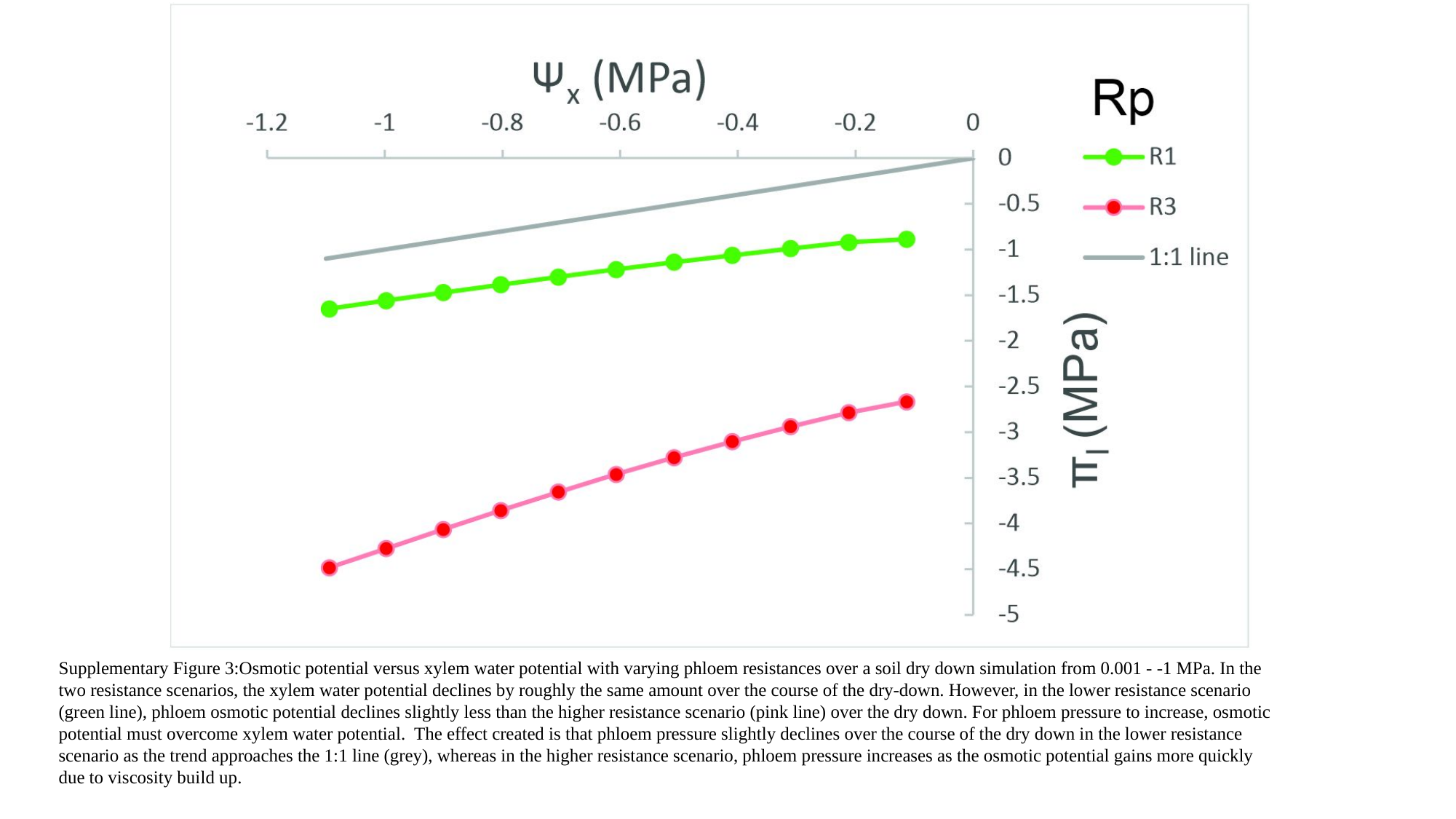

Supplementary Figure 3:Osmotic potential versus xylem water potential with varying phloem resistances over a soil dry down simulation from 0.001 - -1 MPa. In the two resistance scenarios, the xylem water potential declines by roughly the same amount over the course of the dry-down. However, in the lower resistance scenario (green line), phloem osmotic potential declines slightly less than the higher resistance scenario (pink line) over the dry down. For phloem pressure to increase, osmotic potential must overcome xylem water potential. The effect created is that phloem pressure slightly declines over the course of the dry down in the lower resistance scenario as the trend approaches the 1:1 line (grey), whereas in the higher resistance scenario, phloem pressure increases as the osmotic potential gains more quickly due to viscosity build up.

## Slide 5
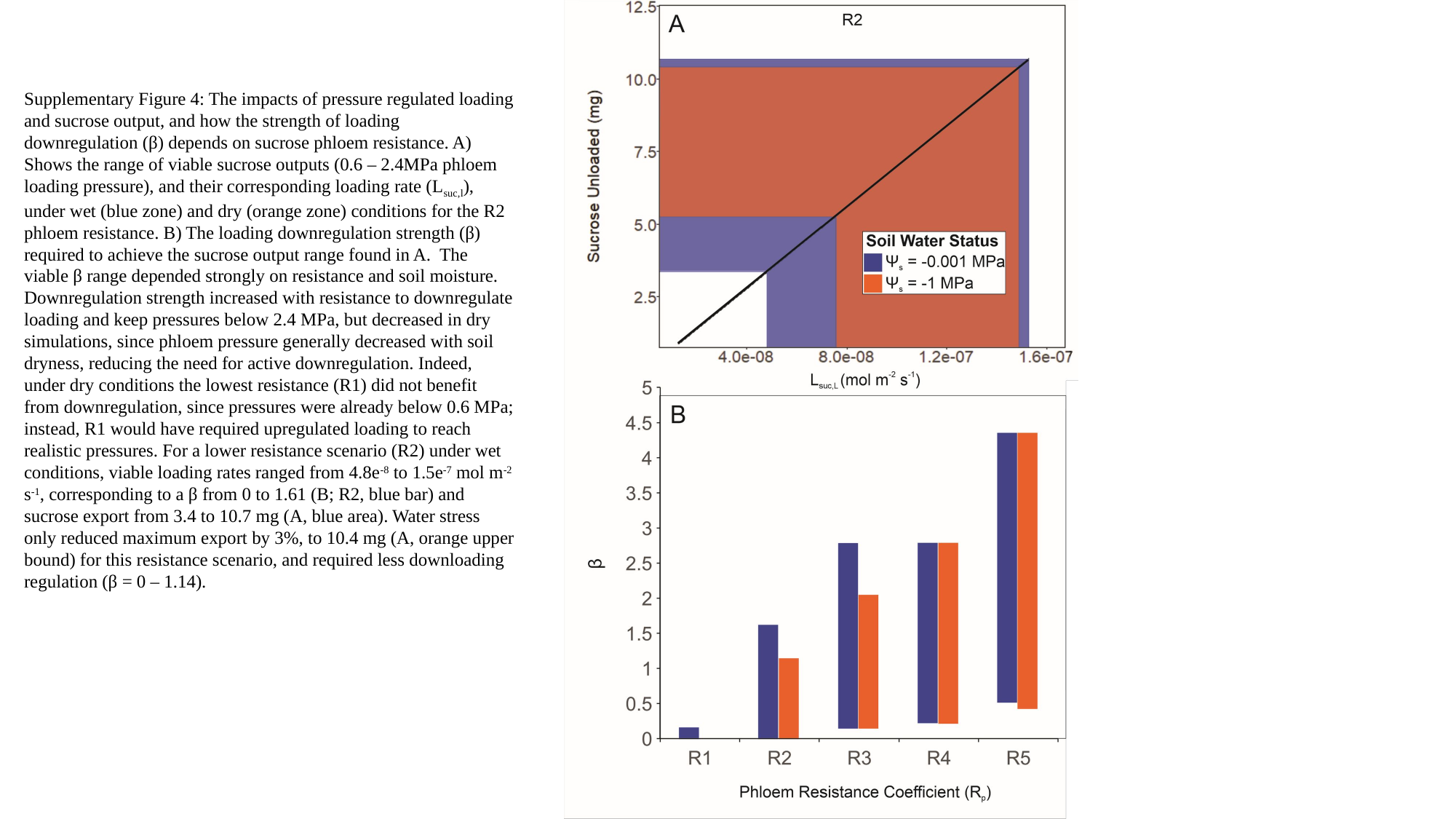

Supplementary Figure 4: The impacts of pressure regulated loading and sucrose output, and how the strength of loading downregulation (β) depends on sucrose phloem resistance. A) Shows the range of viable sucrose outputs (0.6 – 2.4MPa phloem loading pressure), and their corresponding loading rate (Lsuc,l), under wet (blue zone) and dry (orange zone) conditions for the R2 phloem resistance. B) The loading downregulation strength (β) required to achieve the sucrose output range found in A. The viable β range depended strongly on resistance and soil moisture. Downregulation strength increased with resistance to downregulate loading and keep pressures below 2.4 MPa, but decreased in dry simulations, since phloem pressure generally decreased with soil dryness, reducing the need for active downregulation. Indeed, under dry conditions the lowest resistance (R1) did not benefit from downregulation, since pressures were already below 0.6 MPa; instead, R1 would have required upregulated loading to reach realistic pressures. For a lower resistance scenario (R2) under wet conditions, viable loading rates ranged from 4.8e-8 to 1.5e-7 mol m-2 s-1, corresponding to a β from 0 to 1.61 (B; R2, blue bar) and sucrose export from 3.4 to 10.7 mg (A, blue area). Water stress only reduced maximum export by 3%, to 10.4 mg (A, orange upper bound) for this resistance scenario, and required less downloading regulation (β = 0 – 1.14).
