# Supplementary material for: Coordination Between Phloem Loading and Structure Maintains Carbon Transport Under Drought
Source: Front Plant Sci. 2022 Feb 17;13:787837. doi: 10.3389/fpls.2022.787837 (PMC8891486; doi:10.3389/fpls.2022.787837)
Supplement: Supplementary file 2 [file Table_1.DOCX]

Support Information Table 1. References used to categorize species by loading type used in Fig. 1. Rse and Rsel are the calculated total sieve element resistance and sieve element length, respectively (data from Liesche et al. 2017).

|  |  |  | |  | |  |  |
| --- | --- | --- | --- | --- | --- | --- | --- |
|  | Species (resistance symbol) | Loading Type | Reference for Loading Type | | Selected Rse (1/µm^3^) | | Selected Rsel (µm) |
|  | *Gossypium barbadense* (R5) | Active | Muller et al. 2014 | | 0.636 | | 375 |
|  | *Ricinus communis* (R3) | Active | Tamas and Davies 2016 | | 0.0566 | | 255 |
|  | *Liriodendron chinense* (R2) | Active | Fu et al. 2011 | | 0.0495 | | 457 |
|  | *Magnolia denudata* | Active | Fink et al. 2018 | |  | |  |
|  | *Robinia pseudoacacia* (R4) | Active | Gamalei 1989 | | 0.0822 | | 180 |
|  | *Vitis vinifera* (R1) | Active | Davies et al. 1999 | | 0.0240 | | 500 |
|  | *Fraxinus americana* | Active Polymer Trap | Gamalei 1989; Fu et al. 2011 | |  | |  |
|  | *Castanea dentata* | Passive | Gamalei 1989 | |  | |  |
|  | *Fagus sylvatica* | Passive | Gamalei 1989; Fu et al. 2011 | |  | |  |
|  | *Juglans cinerea* | Passive | Rennie and Turgeon 2003 | |  | |  |
|  | *Juglans nigra* | Passive | Rennie and Turgeon 2003 | |  | |  |
|  | *Ligustrum lucidum* | Passive | Gamalei 1989 | |  | |  |
|  | *Populus deltoides* | Passive | Fu et al. 2011 | |  | |  |
|  | *Prunus persica Batch.* | Passive | Rennie and Turgeon 2003 | |  | |  |
|  | *Pyrus malus* | Passive | Fu et al. 2011 | |  | |  |
|  | *Quercus faginea* | Passive | Rennie and Turgeon 2003 | |  | |  |
|  | *Sabal palmetto* | Passive | Gamalei 1989 | |  | |  |
|  | *Salix nigra* | Passive | Fu et al. 2011 | |  | |  |
|  | *Salix pentandra* | Passive | Fu et al. 2011 | |  | |  |
|  | *Spathodea campanulata* | Passive | Gamalei 1989 | |  | |  |
|  | *Tectona grandis* | Passive | Gamalei 1989 | |  | |  |
|  | *Tilia americana* | Passive | Rennie and Turgeon 2003 | |  | |  |
|  | *Ulmus americana* | Passive | Gamalei 1989 | |  | |  |
|  | *Quercus faginea* | Passive | Rennie and Turgeon 2003 | |  | |  |
|  | *Sabal palmetto* | Passive | Gamalei 1989 | |  | |  |
|  | *Salix nigra* | Passive | Fu et al. 2011 | |  | |  |
|  | *Salix pentandra* | Passive | Fu et al. 2011 | |  | |  |
|  | *Spathodea campanulata* | Passive | Gamalei 1989 | |  | |  |
|  | *Tectona grandis* | Passive | Gamalei 1989 | |  | |  |
|  | *Tilia americana* | Passive | Rennie and Turgeon 2003 | |  | |  |
|  | *Ulmus americana* | Passive | Gamalei 1989 | |  | |  |
|  |  |  | |  | |  |  |
|  |  |  | |  | |  |  |
|  |  |  | |  | |  |  |
|  |  |  | |  | |  |  |
|  |  |  | |  | |  |  |
|  |  |  | |  | |  |  |
|  |  |  | |  | |  |  |
|  |  |  | |  | |  |  |
|  |  |  | |  | |  |  |
|  |  |  | |  | |  |  |
|  |  |  | |  | |  |  |
|  |  |  | |  | |  |  |
|  |  |  | |  | |  |  |
|  |  |  | |  | |  |  |
|  |  |  | |  | |  |  |
|  |  |  | |  | |  |  |
|  |  |  | |  | |  |  |
